# High rate of safety and fraud issues in commercially available cinnamon

**DOI:** 10.1038/s41538-025-00485-w

**Published:** 2025-07-05

**Authors:** M. Ghidotti, M. Behr, D. Pietretti, M. Jiménez, L. Garlant, S. Papoci, M. B. de la Calle Guntiñas

**Affiliations:** European Commission, Joint Research Centre (JRC), Geel, Belgium

**Keywords:** Chemistry, Analytical chemistry, Chemical safety

## Abstract

The consumption of cinnamon, and next to it its price, have increased the last years making of this spice an attractive target for fraudsters. This work presents the outcome of a study in which 104 cinnamon samples purchased at retailers in EU countries, have been investigated. The study showed that a high share of samples, 66.3%, either did not fulfil quality criteria set by international standards, were not compliant with European food safety legislation, were suspicious of fraud, or could be toxic for children due to a high content of coumarin. Substitution of Ceylon by Cassia cinnamon, so far the most recognised type of fraud, was not the problem most frequently detected in this study. The information provided by several techniques, namely Energy Dispersive X-Ray Fluorescence, Head Space-Gas Chromatography-Mass Spectrometry, q-PCR, and Termogravimetric Analyses, was needed to cover the full range of irregularities detected in the study.

## Introduction

The European Parliament resolution of 14 January 2024 on the food crises, fraud in the food chain and control thereof^1^, states that spices are one of the food commodities most frequently adulterated. In 2023, cinnamon was the fifth most imported spice in the European Union, primarily from Vietnam^2^, after ginger, paprika, pepper and turmeric. Currently, the main cinnamon producers in the world are China, Vietnam, Indonesia, Sri Lanka, and Madagascar, all together accounting for more than 99% of the world’s production^3^; in particular, China and Vietnam produce about 89% of the world’s cinnamon.

Cinnamon demand is expected to grow the next few years, with a global compound annual growth rate (CAGR) of 6.4–12.4%, Europe following the global trend^4^. Cinnamon whose cultivation dates back to 3000 BC, has always had two main uses, culinary and medicinal^5,6^. Nowadays, cinnamon is a key ingredient in a growing number of new products (e.g. dairy products, beverages)^7^, and some of its medicinal applications, are growing in popularity throughout the world^8^. Consumption of cinnamon will very likely increase, following the trend to eat healthy sustainable diets containing flavour-intensifying ingredients^9^, as recommended by the EAT-Lancet Commission^10^. The increase in demand has already triggered an increase in the price of cinnamon in Europe at an annual rate of 10% since 2017^7^.

Cinnamon originated from the genus *Cinnamomum*, which belongs to the *Lauraceae* family and includes about 250 species spread over South East Asia, China and Australia, many of which are aromatic and flavouring. The highest quality cinnamon is *Cinnamomum zeylanicum* (syn. *C. zeylanicum* Blume) (Ceylon cinnamon), native to Sri Lanka, and produced also at The Seychelles, Madagascar and India in small quantities. It is pale in colour and has a mildly sweet flavour. There are four major grades of Ceylon cinnamon based on the diameter of the cinnamon sticks, namely Alba, Continental, Mexican and Hamburg. Among these, Alba with a 6-mm diameter is the highest quality^11^.

Cassia is a similar but stronger-flavoured spice of lower quality native of Myanmar (Burma). Cassia varieties, referred to as “cassia cinnamons”, include Chinese cassia (*C. cassia* syn. *C. aromaticum*) from China and Vietnam, Indonesian (*C. burmanii*), from Sumatra and Java, Vietnamese cinnamon (*C. loureiroi*) and Indian cassia (*C. tamala*) from the north-eastern India and Myanmar^12^. From a safety point of view Cassia cinnamon contains coumarin, a liver toxic compound with a Tolerable Daily Intake (TDI) of 0.1 mg kg^−1^ bodyweight^13^, which is absent or present in very low amounts in Ceylon cinnamon.

The increasing demand of cinnamon may challenge the supply chain fuelling malpractices and fraudulent activities. The significant price difference between Ceylon and Cassia cinnamon, the former selling at approximately twice the price of the latter, adds to the motivations for fraudulent practices. These practises may include substitution (partial or complete) of Ceylon cinnamon by Cassia cinnamon in ground cinnamon, or adulteration of both Ceylon and Cassia cinnamon bark with other parts of the plant (e.g., root, leaves, flowers, seeds), or with other bulking agents.

Cinnamon marketed in the EU must comply with several regulations (Regulation (EC) No 178/2002; Regulation (EU) No 1169/2011), and precisely Commission Regulations (EU) 2023/915 and (EC) No 1333/2008, which respectively set a maximum level of 2.0 mg lead per kg and 150 mg of sulphur dioxide-sulphites per kg in cinnamon. Additionally, Regulation (EC) No 1334/2008 sets maximum levels of coumarin ranging from 5 mg kg^−1^ in desserts to 50 mg kg^−1^ in traditional and/or seasonal bakery products labelled as containing cinnamon. However, no specific provisions apply to coumarin naturally present in cinnamon. Non-compliance with maximum allowed levels of lead have been reported at the Rapid Alert System for Food and Feed (RASFF) of the European Commission^14^ and by the Food and Drug Administration (FDA)^15^.

As indicated in a review recently published on analytical methods for cinnamon authentication^8^, most of the studies conducted so far on that subject, focus on detection of substitution of Ceylon cinnamon by Cassia cinnamon, based on the analyses of markers such as coumarin, that are present in very different concentrations in both species. A few studies focus on the substitution of cinnamon by other spices. However, a holistic study on commercially available cinnamon integrity, including not only detection of substitution practices using different plants, but also substitution of bark with different part of the cinnamon plant, safety issues, such as high content of contaminants, and poor quality according to international standards^16,17^, is missing.

This work describes the outcome of a study carried out on more than hundred commercially available cinnamon samples purchased at retailers and specialised shops in eleven countries of the European Union, with the aim of detecting and identifying possible fraudulent practises, and non- compliances with existing European legislation and international standards. Four methods have been used in the analyses of the samples, namely Energy Dispersive x-Ray Fluorescence (EDXRF), Head Space-Gas Chromatography-Mass Spectrometry (HS-GC-MS), Thermogravimetric Analyses (TGA) and quantitative Polymerase Chain Reaction (q-PCR) (applied only to ground samples), each one of them providing information about different types of adulteration and/or lack of quality of cinnamon^8,18^.

## Results

### Low quality according to International Standards regarding the total ash content

TGA was used to determine the total ash content in all test materials, to detect samples that do not fulfil the quality requirements of ISO 6539 (*C. zeylanicum*) and ISO 6538 (Cassia cinnamons). Test materials that do not fulfil those requirements are not necessarily suspect of adulteration but lack the required quality to be compliant with international standards. The maximum allowed total ash content is based on two parameters, botanical species (Ceylon cinnamon or Cassia cinnamon), and within those groups, geographical origin. The maximum limit for total ash content in Ceylon cinnamon are 5 and 7% for Sri Lankan, and Seychelles and Madagascan types, respectively. For Cassia cinnamons, the limits are 4, 4.5 and 5% for Chinese, Vietnamese and Indonesian types, respectively. This means that this parameter can only be evaluated in samples for which both, botanical and geographical origin are declared. That information was provided for 28 samples, out of which six did not fulfil the quality criteria. One Cassia cinnamon sample without geographical origin information, had a total ash content higher than 5%. This means that 20.7% of the samples providing enough information on the labels, and 5.8% of the total amount of samples analysed, does not fulfil international quality standards requirements. Nine Ceylon cinnamon samples with no information on geographical origin in the label had a total ash content between 5 and 7% and would lack the required quality unless they would come from Madagascar or Seychelles. The compliance with quality standards would be difficult to assess in samples containing mixtures of several botanical and geographical origins, because it would depend on the proportions used.

### Samples non-compliant with safety legislation

Ten samples (2 Ceylon cinnamons, 2 cassia and 6 without botanical origin information) were not compliant with Commission Regulation (EU) 2023/915, that sets a maximum level of Pb in bark species of 2.0 mg kg^−1^, corresponding to 9.6% of the samples analysed. As mentioned in the introduction, several alerts made in RASFF and by the FDA, reflect this problem. Some alerts in RASFF report on high levels of Hg; unfortunately, the limits of quantification (LOQs) of the EDXRF method used in this study are too high to screen compliance with legislation for Hg. The FDA has also reported high levels of Cr in cinnamon samples. In this study, nineteen samples (11 Ceylon cinnamons, 2 Cassia cinnamons, and 6 without information about botanical origin) were outliers due to a high Cr level, ranging from 2 to 20 mg kg^−1^. The European legislation on contaminants does not have maximum limits for Cr because the TDI for Cr in food set by the European Food Safety Authority (EFSA) is 0.3 mg kg^−1^ body weight^19^, a value not exceeded by any of the groups of population included in the opinion study. Information about LOQs, and distribution of results according to the elements analysed by EDXRF is given in Table 1.

**Table 1:** Summary of the LOQs, and expanded uncertainties (*k* = 2) of the EDXRF and HS-GC-MS-based methods used to carry out the elemental and volatile analysis, respectively, and of the concentration distribution among samples of the elements and volatile compounds included in this study using the mention methods

Compound/Element	LOQ	U (*k* = 2) (%)	Percentile 25	Percentile 50 (Median)	Percentile 75	Mean	C.I. (95%)	Statistical outliers	Strong outliers
Pinene	0.03	30.0	6.84	15.99	47.7	68.1	0.20–101	101–200 (*n* = 5)	200–1064 (*n* = 7)
Limonene	0.18	27.8	1.73	18.7	44.3	42.4	0.62–99.2	99.2–150 (*n* = 4)	150–439 (*n* = 7)
Benzyl alcohol	1.24	19.6	ND^a^	20.4	43.7	45.4	ND–100	100–250 (*n* = 6)	567 (*n* = 1)
**Camphor**	**2.34**	**28.7**	**<LOQ**	**4.55**	**9.74**	**28.8**	**<LOQ–20.6**	**20.6–60** (*n* = **7**)	**90–871** (*n* = **6**)
Hydrocinnamaldehyde	0.76	16.0	142	326	420	316	21.9–654		800–1279 (*n* = 2)
**Anethole**	**4.36**	**26.9**	**ND**^a^	**<LOQ**	**<LOQ**	**5.04**	**ND–5.50**	**5.50–20** (*n* = **10**)	**20–183** (*n* = **3**)
**Eugenol (Ceylon c.)**	**2.60**	**4.4**	**17.1**	**49.4**	**144**	**119**	**6.18–322**	**322–467** (*n* = **4)**	**626** (*n* = **1**)
**Eugenol (Cassia C.)**			**<LOQ**	**<LOQ**	**5.19**	**12.6**	**<LOQ–7.74**		**38.4–125** (*n* = **2**)
**Caryophyllene**	**0.52**	**26.0**	**56.7**	**227.5**	**1272**	**822**	**19.0–3055**	**3055–3304** (*n* = **1**)	**4000–5158** (*n* = **3**)
**Coumarin (Ceylon c.)**	**1.50**	**26.2**	**2.72**	**3.49**	**5.55**	**6.81**	**1.88–9.44**		**101** (*n* = **1**)
**Coumarin (Cassia c.)**			**182**	**269**	**429**	**371**	**1.68–680**		**800–1042** (*n* = **2**)
**Cinnamyl acetate**	**1.49**	**10.2**	**0.84**	**8.14**	**69.1**	**97.35**	**ND–162**	**162–400** (*n* = **7**)	**400–751** (*n* = **9**)
**Eugenol acetate**	**0.52**	**51.4**	**ND**^a^	**<LOQ**	**<LOQ**	**<LOQ**	**ND–**<**LOQ**	**LOQ–0.60** (*n* = **2**)	**1.00** (*n* = **1**)
**Caryophyllene oxide**	**0.51**	**8.2**	**1.21**	**6.65**	**17.4**	**20.5**	**ND–38.9**	**40–55** (*n* = **3**)	**60.0–151** (*n* = **9**)
**Benzyl benzoate (Ceylon cinnamon)**	**0.42**	**10.96**	**61.2**	**139**	**334**	**298**	**21.95–716**	**716–1000** (*n* = **2**)	**1000–1894** (*n* = **2**)
**Benzyl benzoate (Cassia cinnamon)**			**3.60**	**6.40**	**14.6**	**18.2**	**1.34–19.6**		**167** (*n* = **1**)
Cinnamaldehyde	1.56	21.7	11,378	12,671	14,258	12,887	7639–17,475	4751–7638 (*n* = 2) 17,475–20,731 (*n* = 2)	24,287 (*n* = 1)
**Al**	**310**	**7**	**<LOQ**	**<LOQ**	**463**	**374**	**<LOQ–1016**		**1050–2512** (*n* = **8**)
As	0.18	7	ND^a^	ND^a^	ND^a^	ND	ND^a^		
Ba	6.4	6	30.5	45.1	63.5	48.1	7.91–102	116 (*n* = 1)	
Br	0.35	19	0.66	1.26	2.41	6.18	<LOQ–4.09	4.09–20 (*n* = 7)	20.0–143 (*n* = 11)
Ca	118.4	4	9632	12,572	15,383	13,186	6221–23,349	25,945 (*n* = 1)	47,312 (*n* = 1)
Cd	0.56	9	ND^a^	<LOQ	<LOQ	<LOQ	ND-0.76		
Cl	222^b^	5	<LOQ	284	482	320	<LOQ-797		
**Cr**	**0.6**	**12**	**<LOQ**	**0.70**	**1.12**	**1.51**	**<LOQ–1.79**	**1.79–5.0** (*n* = **14**)	**5.0–19.9** (*n* = **5**)
Cu	2.8	15	3.87	6.66	9.09	6.87	2.92–13.2		
**Fe**	**9.4**	**10**	**33.9**	**66.9**	**326**	**257**	**<LOQ–764**	**899–1133** (*n* = **6**)	**1825–2213** (*n* = **2**)
Hg	0.32	7	<LOQ	<LOQ	<LOQ	<LOQ	<LOQ		
K	1282	4.5	8875	9956	12,293	10,821	5348–17,280	17,701–19,537 (*n* = 5)	
Mg	313^b^	10	546	654	812	679	327–1165		
Mn	1.78	12	142	206	279	230	32–452	579–680 (*n* = 2)	
Mo	0.38	19	<LOQ	<LOQ	0.47	<LOQ	ND–0.99	1.16–2.58 (*n* = 8)	
Na	375	9.5	<LOQ	<LOQ	591	468	ND–1041	1163–1390 (*n* = 6)	
Ni	0.57	11	0.75	1.15	2.15	1.85	LOQ–4.09	4.60–8.22 (*n* = 11)	
P	671	8	<LOQ	680	798	718	<LOQ–1059	1110–1345 (*n* = 6)	
**Pb**	**0.75**	**11**	**<LOQ**	**<LOQ**	**0.99**	**0.97**	**<LOQ–1.52**	**1.53** (*n* = **1**)	**1.96–5.11** (*n* = **11**)
Rb	2.76	4	17.9	21.9	28.1	23.6	6.82–41.25	46.48–53.54 (*n* = 2)	
**S**	**30**	**15**	**936**	**1096**	**1290**	**1342**	**752–1817**	**1856–1993** (*n* = **3**)	**3003–5590** (*n* = **10**)
Se	0.37	13	ND^a^	ND^a^	<LOQ	0.53	ND–0.60	0.75–2.75 (*n* = 12)	3.67–5.83 (*n* = 6)
***Si***	***2370***	***5***	**<LOQ**	**<LOQ**	**<LOQ**	**<LOQ**	**<LOQ**		**2604–3606** (*n* = **2**)
Sr	1.19	4	68.6	88.3	105	89.1	28.5–154	233–235 (*n* = 2)	
***Ti***	***12.3***	***1***	**ND**	**ND**	**23.4**	**<LOQ**	**ND–73.5**	**92.4–96.9** (*n* = **2**)	**202–231** (*n* = **2**)
Zn	6.5^b^	3	11.5	13.8	16.8	14.7	<LOQ–24.9	26.4–34.8 (*n* = 4)	
**Zr**	**10.24**^b^	**7**	**ND**	**ND**	**<LOQ**	**<LOQ**	**ND–**<**LOQ**		**12.7–33.51** (*n* = **7**)

All concentrations in mg kg^−1^. Elements and compounds in bold are markers to be used for detection of adulteration and/or safety issues

^a^*ND* Not detected

^b^Lowest mass fraction in the CRMs/RMs available in our laboratory. The LOQ could be lower than that

Thirteen samples were outliers due to a high sulphur content (between 1.4 and 4 times the median of the results for the sulphur concentrations covered by the 95% confidence interval), Table 1. Those samples could be not compliant with Commission Regulation (EU) 1129/2011 on food additives that sets 150 mg kg^−1^ as maximum level for sulphur dioxide-sulphites in cinnamon. Non-compliance of cinnamon with that legislation due to sulphites has been reported in RASFF^20^. Nevertheless, the method used in this study measures the total content of sulphur; in the frame of control activities those samples should be analysed with a method allowing the specific quantification of sulphur dioxide and sulphites.

The ranges for coumarin in the samples analysed are given in Table 1. As expected, the concentration of coumarin in Cassia cinnamon is much higher than in Ceylon cinnamon. Two sample were outliers due to the high coumarin content. However, it is difficult to evaluate the compliance of some of the Cassia cinnamon samples with Regulation (EC) No 1334/2008 setting maximum levels of coumarin in desserts and bakery products, because it depends on the quantity of cinnamon used in the preparation of the final product. Accordingly, compliance with legislation must be tested in the final product. Information about compliance with the TDI set by EFSA^13^, can be found later on in the Discussion section.

### Substitution of Ceylon cinnamon by Cassia cinnamon

EDXRF was used as targeted screening method to detect substitution of Ceylon cinnamon by Cassia cinnamon. A previous study^18^ showed that in general, and with the exception of manganese, the elemental content of cassia cinnamon is lower than that of Ceylon cinnamon, and for this reason, the elemental profile determined by EDXRF can be used to detect substitution of Ceylon by cassia cinnamon. The results obtained were combined with multivariate analyses and modelling techniques, to identify Ceylon cinnamon suspicious of having been substituted by cassia cinnamon or by other species/materials, as described later.

**Fig. 1:**
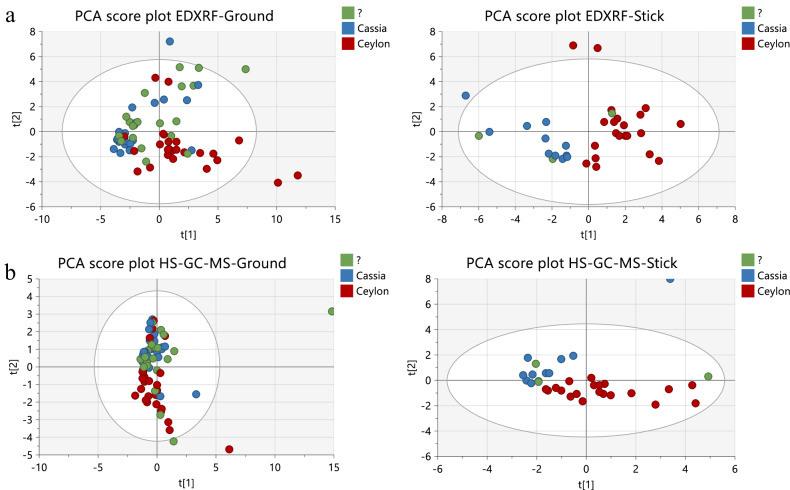
PCA score plots for ground and stick cinnamon. PCA score plots for **a** Ground and stick cinnamon samples based on elemental profile determined by EDXRF, and **b** Ground and stick cinnamon samples based on HS-GC-MS.

EDXRF was able to detect four test ground materials labelled as Ceylon cinnamon that had been substituted totally or partially by cassia cinnamon. The four samples were projected among cassia samples in the two Principal Component Analysis (PCA) score plots created using the two techniques, respectively (Fig. 1a). A fifth sample without any indication on the label about its botanical origin, but labelled as produced in Sri Lanka, was also identified as cassia. The label claims of Sri Lankan origin could be considered as an intention to deceit the consumer.

**Fig. 2:**
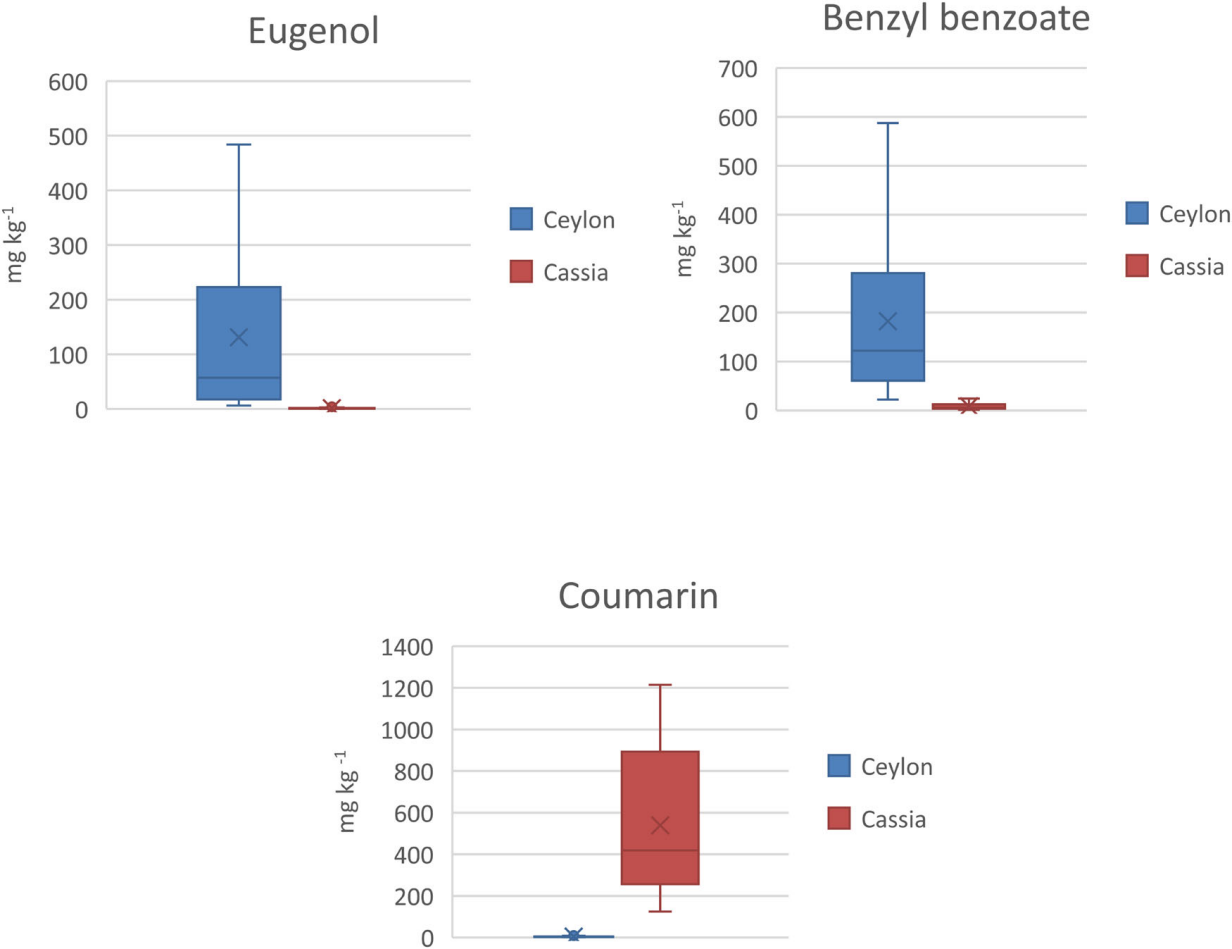
Box and whisker plots for Ceylon and cassia cinnamon biomarkers. Box and whisker plots for eugenol, benzyl benzoate and coumarin in Ceylon and cassia cinnamon test materials according to their label.

HS-GC-MS was used to confirm the information obtained by the screening method. Ceylon cinnamon can be identified by the presence of eugenol and benzyl benzoate, both absent or present in very low amounts in cassia cinnamon, while coumarin, present in cassia cinnamon, is either absent or present in small amounts in Ceylon cinnamon^21^. Figure 2 shows the box and whisker plot for eugenol, benzyl benzoate and coumarin in Ceylon cinnamon and cassia. The four cases of suspicion of substitution of Ceylon cinnamon by cassia were confirmed by HS-GC-MS, based on the content of the mentioned samples in eugenol, benzyl benzoate and coumarin. PCA score plots for ground and stick samples based on a selection of 14 volatile compounds (pinene, limonene, benzyl alcohol, camphor, hydrocinnamaldehyde, anethole, cinnamaldehyde, eugenol, caryophyllene, coumarin, cinnamyl acetate, eugenol acetate, caryophyllene oxide, benzyl benzoate) determined by HS-GC-MS, are shown in Fig. 1b. Figure 1b, shows that four Ceylon cinnamon samples were plotted among the cassia cinnamon samples. The 14 volatile compounds were selected based on a previous study^18^. Information about the LOQ of the compounds analysed and their distribution in the tested samples is given in Table 1.

### Substitution of cinnamon bark by other parts of the plant and or by other spices

The selected volatile compounds would allow not only the detection of substitution of Ceylon cinnamon with Cassia cinnamon, but also to detect the substitution of bark with other parts of the cinnamon plant (such as leaves, flowers, roots, and seeds) based on the difference in relative abundances of the selected compounds in different parts of the plant^22^. Cinnamaldehyde is the dominating compound in the bark, eugenol is the most abundant volatile compound in Ceylon cinnamon leaves, 70–95% in comparison with the 5–10% in bark; camphor is the most abundant volatile in the roots of the plant; cinnamyl acetate, is the predominant compound in fruit, flowers, and fruit stalks of Ceylon cinnamon^22^.

Six test materials (5 Ceylon cinnamon and one without information on geographical indication) had high levels of camphor (2-bornanone), (between 5 and 40 times the highest content covered by the 95% confidence interval, Table 1). In cinnamon, camphor is present in the roots of the plant and therefore, an explanation for the high camphor concentration could be that bark has been partially substituted by the root of the plant. Another explanation could be that Ceylon cinnamon has been substituted by *C. camphora*, another cinnamon species. Samples high in camphor were also high in one or several of the following elements Al, Si, Ti, Fe, Cr, and Zr, when analysed by EDXRF. Twelve more samples contained camphor at concentrations slightly higher than those covered by the 95% confidence interval, some of them also characterised by relatively high levels of Al, Si, Ti, Fe and/or Zr. Those samples were not considered as suspicious of substitution of the bark because the relatively high levels of camphor could be due to cross-contamination.

Methyl esters of some fatty acids such as myristic, palmitic, linoleic, oleic, and stearic acids were detected in eight samples. Methyl esters of these fatty acids could be formed by partial methylation of the free acids by the internal standard solution containing methanol during the extraction of volatiles that occurred at 150 °C for 15 min. Seeds are rich in fatty acids^23^ and the presence of detectable amounts of some fatty acids in the mentioned samples would suggest that they contain not only cinnamon bark but also seeds. Quantification of those compounds was not carried out due to the extremely elevated price of the standards for those substances. The identification was done through their respective masses.

Eugenol acetate could be used to detect the adulteration of cinnamon with clove (Syzygium aromaticum)^24^ and pinene, limonene and anethole with pepper (Piper nigrum)^25,26^. In most of the cinnamon samples the content of anethole was below the LOQ and even the limit of detection (LOD) of the method. Eleven samples had levels of anethole that were outliers among the anethole results. For eight of the samples, the presence of anethole (between 1 and 3 times the LOQ of the method) could be due to cross contamination. For the remaining three samples, the anethole content is so high (14–42 times the LOQ of the method), that it would be difficult to explain by cross contamination. Some of the samples with high anethole content were also high in pinene and limonene content. Out of the three, the one with the highest anethole content was sold as stick, without information about botanical or geographical origin, although a visual observation indicated that it was Cassia cinnamon. One of the two ground cinnamon samples had also a eugenol acetate content that was an outlier among the eugenol acetate results (most of them below the LOQ of the method).

Quantitative Polymerase Chain Reaction was specifically used to detect the presence of clove and pepper in ground cinnamon, type of adulteration previously reported in literature^27,28^. Two q-PCR methods were developed in-house and validated to quantify clove and black pepper in the ground samples included in the study. Neither pepper nor clove could be quantified by q-PCR in any of the analysed samples (LOQ of 2.5% m:m and 3.8% m:m, respectively). However, *Allium cepa* (onion), was detected by q-PCR in the ground sample with high levels of anethole and eugenol acetate, onion contains anethole. This information can only be considered as indicative because the q-PCR methods had not been developed and fully validated for the detection of onion in cinnamon. No explanation could be found for the extremely high anethole content in the stick cinnamon sample.

On top of the anomalies related to the contents of anethole and eugenol acetate described earlier in this chapter, 24 samples were outliers for the composition of some of the volatile compounds quantified by HS-GC-MS, which points at the substitution of the bark by other spices or even parts of the cinnamon plant. Some samples are outliers with a too high content of eugenol, which is the most abundant volatile compounds in Ceylon cinnamon leaves; other samples are outliers with a high content of cinnamyl acetate, predominant compound in fruit, flowers, and fruit stalks of Ceylon cinnamon; some others are outliers with a high content of caryophyllene oxide that is particularly present in Ceylon cinnamon flowers, and other in caryophyllene present in the fruit. Some samples have a low hydrocinnamaldehyde content with less than one third the median of all samples, while other three have 3–4 times more than the median. With the exception of five samples (3 Ceylon cinnamons and 2 without information about botanical origin), the remaining ones had already been flagged as suspicious for other reasons. The four exceptions were labelled as “Suspicious?” and would need further investigation, Supplementary Table 1. The same label was used with three Ceylon cinnamon samples with high levels of S as only outlying component and in which no other anomaly was detected.

q-PCR analyses highlighted the presence of rice (Oriza Sativa) in one sample in amounts high enough to exclude cross-contamination. Six samples (three Ceylon cinnamon, one cassia, one mixture of Ceylon and cassia, and one without botanical origin information) were flagged as suspicious due to the presence of a large number of extraneous species, among others onion, fenugreek (*Trigonella foenum-graecum*), white mustard (*Sinapis alba*) and rice. Only two out of the seven samples considered suspicious by qPCR were not flagged as suspicious and/or non-compliant by any of the other used techniques. Then again, these findings would need further investigation with methods fully validated for the detection/quantification of the respective species in cinnamon using specific primers, before been considered conclusive. Validation of a qPCR method for each one of the different species suspect of having been fraudulently added to cinnamon, goes beyond the purpose of this study, which is to detect and identify possible fraudulent practises in commercially available cinnamon.

## Discussion

The main outcome of this study is that only 33.7% of the 104 samples analysed were free of any suspicion of fraud, lack of quality or safety concerns, Table 2. The lowest rate of non-suspicious samples was found among the Ceylon cinnamon samples, only 5%, all of them commercialised as sticks. The higher rate of irregularities among Ceylon cinnamon samples is logical because Ceylon cinnamon is about twice as expensive as Cassia cinnamon, and therefore the economic benefit for fraudsters is higher.

**Table 2:** Summary of the findings obtained on commercially available cinnamon samples

Type of irregularity	Ceylon cinnamon (%)	Cassia (%)	? (%)	Total (%)
*Rate of samples legally non-compliant with Pb maximum limits, lacking quality, suspicious of adulteration, outliers due to unusual chemical composition*				
Legally non-compliant, Pb >2 mg kg^−1^ (*n* = 10)	4.5	11.8	14.0	9.6
Potentially hazardous for children due to high levels of coumarin (*n* = 13)	–	35	48.9	29.8
Substitution of Ceylon cinnamon by cassia (*n* = 4)	9.1	–	–	3.8
Substitution of Ceylon cinnamon by cinnamon camphora or by cinnamon roots (*n* = 6)	11.4	–	2.3	5.8
Total ash content not compliant with international standards, and/or cross-contamination with many different species (*n* = 10)	13.6	11.8	4.7	9.6
Suspicious (strong outliers for several techniques based on composition information) (*n* = 24)	31.8	11.8	18.6	23.1
Suspicious? (statistical outliers due to unusual composition, further investigation needed) (*n* = 8)	13.6	–	2.3	7.7
*Non suspicious samples*				
Samples not covered by any of the groups above and found non-suspicious (*n* = 40)	4.8	58.8	58.1	38.5
Ceylon cinnamon from Madagascar not evaluated (*n* = 5)	11.4	–	–	4.8

A significant number of samples raised safety concerns. Ten samples, representing 9.6%, did not comply with the legislation for contaminants in food due to a content of lead higher than 2 mg kg^−1^. The rate of non-compliant samples for high lead content is even higher, 14%, among samples without botanical origin indication. In the scientific opinion of EFSA on lead in food^29^, it is stated that lead has potential neurodegenerative effects and margins of exposures were such that the possibility of an effect from lead in some consumers, particularly in children from 1 to 7 years of age, cannot be excluded. Thirteen samples, 13.6%, were outliers due to a high content of sulphur, and would be potential non-compliant samples with maximum limits for sulphur dioxide-sulphites.

Another type of safety concern is due to coumarin in Cassia cinnamon. Although the compliance or not with legislation can only be tested in the final products containing cinnamon as an ingredient, some discussion needs to be done keeping in mind the TDI of 0.1 mg kg^−1^ bodyweight set by EFSA^13^. A teaspoon of cinnamon can weight around 5 g. The TDI would have been surpassed by children aged between 6 and 18 months (weight between 7 and 10 kg) consuming in one day 0.5 g (one eighth of a tea spoon) of the Cassia sample with the highest coumarin content. The limit would be surpassed for the same population consuming about 1 g of the thirteen Cassia cinnamon samples with a coumarin content higher than 838 mg kg^−1^. Five out of those 13 samples are not suspicious of adulteration and are compliant with existing legislation (marked with a yellow background in Supplementary Table 1). Some studies propose the use of up to 6 g day^−1^ of cinnamon, for up to 4 months to reduce fasting plasma glucose levels^30^. The consumption of milk shakes and smoothies has increased in recent years, and many recipes include one or one and half teaspoon cinnamon as flavouring agent. The use of one teaspoon of the Cassia cinnamon with the two highest coumarin content included in this study, would have passed the TDI value even for adults with a weight of up to 80 kg and 60 kg, respectively. Other 31 samples (21 of the cinnamon samples without botanical information, 6 Cassia cinnamons and 1 of the samples labelled as Ceylon cinnamon but found to be totally or partially substituted by Cassia cinnamon), would have the same effect if the milk shake/smoothie had been consumed by children younger than 10 years old (estimated weight 20 kg or less). This means that 29.8% of the total, 35% of the Cassia cinnamon and 48.9% of the samples without botanical origin information in the label, could represent a safety problem for children younger than ten years of age. The number could be higher because the validation of the HS-GC-MS method used to quantify coumarin did not include recovery studies, and so the amount detected could not correspond to the total content of coumarin. An exhaustive toxicological study goes beyond the scope of this work, which is to propose and validate a method for the detection of fraud in cinnamon. Since it is difficult to know what amount of cinnamon could be used at home for some recipes, cinnamon with high coumarin contents should be labelled accordingly, indicating that the product should not be consumed by children. Methods for the determination of the total content of coumarin in cinnamon should be developed and standardised so that control laboratories can incorporate that type of analyses in their regular controls.

Substitution of Ceylon by Cassia cinnamon, so far the type of fraud more frequently mentioned in the literature^8^, detected in this study in 9% of the samples labelled as Ceylon cinnamon, was outgrown by the number of samples suspicious of other types of substitution, such as bark by roots or Ceylon cinnamon by camphora. Almost 14% of the Ceylon cinnamon samples were strong outliers due to high camphor contents, a compound that is supposed to be present only at trace levels in bark. The high contents in those samples of one or several of the following elements Al, Si, Ti, Fe, Cr, and Zr, normally present at higher concentrations in inorganic materials such as soils, than in organic matrices, supports the hypothesis of substitution of the bark with roots. Substitution of Ceylon cinnamon by camphora would be another feasible explanation but no camphora was found on the market and this hypothesis could not be tested. Most of those samples do not fulfil international quality standards due to a high ash content.

Next to the test items mentioned in the paragraph above, some other samples with normal camphor levels, had contents of Al, Si, Ti, Cr, Fe and Zr higher than those covered by the 95% confidence interval for one or several of the mentioned elements. The origin of those elements could also be the material used to process the cinnamon, for instance to mill it. All the test materials with high levels of Al, Si, Ti, Cr, Fe, Zr and Pb, were commercialised ground, and all of them were considered anomalous also by HS-GC-MS. European legislation for contaminants in food does not provide maximum limits for any of those elements, but the Dutch legislation says that the content of zirconium in food coming from certain zirconium-containing compounds used in food contact materials should not be higher than 2 mg kg^−1^ ^31^. In this study, eight samples had concentrations of zirconium higher than 10 mg kg^−1^, LOQ of the EDXRF method. Some of those samples contained more than 200 mg kg^−1^ titanium, and 2000 mg kg^−1^ aluminium. Those high concentrations were found in samples commercialised as bulk. These findings are in agreement with the results published by Gonçalves et al.^32^, who found Al concentrations higher than 1000 mg kg^−1^, indicating that the Tolerably Weekly Intake (TWI) of 1.0 mg kg^−1^ b.w. week^−1^ for Al set by the FAO/WHO Joint Expert Committee on Food Additives and by EFSA^33^, was closed to be exceeded by the consumption of those products. The consumption of 1.5 g (about one third of a tea spoon) of the cinnamon samples with Al contents higher than 2000 mg kg^−1^ included in this study, would exceed the TWI for children ten years old or younger.

A larger share of samples were strong outliers for several components, including elements and volatiles other than camphor, pointing in the direction of substitution of bark by other parts of the cinnamon plant such as leaves, fruit, seeds and flowers. Those deviations from the normal composition in volatile compounds could represent normal genetic variations^34^, cross-contamination issues that could be normal or due to poor processing practices, or could be the result of fraudulent substitution of the cinnamon bark. Before drawing final conclusions about adulteration rates, mainly based on outlying composition of volatile compounds found in a higher proportion in roots, leaves, flowers, and seeds, Ceylon cinnamon material with full traceability records, including samples from different geographical origins and different cultivars, should be analysed and used as reference to detect adulteration. Otherwise, there is the risk to declare samples of a certain cultivar and varieties as suspicious, because their composition is different from most of the samples available on the European market. Wijesekera and Chichester^34^ published in 1978 a review in which information about cultivars (plantation types) and wild cinnamon varieties are described. Unfortunately, due to the technological limitations at the end of the 70’s, the article lacks full quantitative information. Nevertheless, the review agrees with other articles^22^ that the main volatile components in the different parts of the cinnamon plants are cinnamaldehyde in the bark, eugenol in the leaves, and camphor in the roots, only traces of camphor can be present in the bark. It also needs to be stressed that all the samples referred to in this paragraph, with the exception of four, were suspicious for other reasons.

The exclusive use of samples purchased at supermarkets and small retailers has inherent drawbacks. The study and the conclusions extracted thereof, would have been more robust if genuine samples with full traceability records that can be used as reference of non adulterated products, would have been included. However, in practice this would be extremely difficult. Such samples would have to be treated following the same procedures as those applied to commercially available cinnamon, because processing can introduce changes in the chemical composition of the raw cinnamon, hampering a direct comparison of the raw cinnamon with the final commercial product. The processing and supply chain of spices, including cinnamon, is complex, long, and globalised, and fraudulent manipulations can take place in any stage, making it extremely difficult to have access to samples with full authenticity records.

Eventually, the information provided in Table 1 can help the scientific community and policy makers in setting cutting values for the different cinnamon components, to consider a sample suspicious, and therefore requiring in depth traceability surveillance, for instance based on document control. Nevertheless, it needs to be considered that the recovery of volatile compounds by HS-GC-MS may not be quantitative, and correction factors need to be applied if the results are to be compared with those obtained with other techniques.

Summarising, the type of fraudulent practices detected in commercially available samples in the European market is diverse and cannot be tackled exclusively with one analytical technique; several approaches should be followed to address the problem in a holistic way. However, EDXRF was able to flag as suspicious 87% of all samples lacking quality, those suspicious of substitution by cassia, other plants and different parts of the cinnamon tree, as well as those with anomalous volatile composition. Four non-suspicious samples were flagged as suspicious by EDXRF. The method is the only one among the different analytical approaches used in this study, able to detect samples legally non-compliant due to a high content of Pb, and to flag those with an outlying concentration of sulphur, pointing to the abusive use of SO_2_. For this reason, EDXRF can be considered a suitable screening method. HS-GC-MS can be used as confirmatory method; as expected, it could not detect neither the samples that were suspicious due to a high content of S and/or other elements, nor those not compliant with legislation and international standards due to a high content of Pb, and ash, respectively. HS-GC-MS was able to detect 100% of the remaining suspicious samples; it flagged as suspicious two samples found non-suspicious by the other methods.

Official control laboratories could use EDXRF measurements as screening method, and HS-GC-MS as confirmatory method. Those two techniques were selected because they are fast and hardly require any sample treatment. This does not exclude the use of other techniques widely used for elemental and organic compounds characterisation of samples, such as ICP-MS and LC-MS, respectively. Multivariate analyses in combination with modelling tools facilitates the detection of outlying samples based on their elemental and volatile composition, revealing information that can remain hidden in univariate studies. Not all laboratories are familiar with modelling, but its use is becoming frequent in research and control laboratories. The development, optimisation and validation of qPCR methods require an initial effort to identify specific primers for the different adulterants. However, once developed and validated the technique can provide useful additional information.

The dimension of the problem of irregularities in the cinnamon market is such that it requires attention to be paid to the situation, even more because next to the economic aspect linked to food fraud, several food safety concerns have been raised, and because the global cinnamon consumption is increasing.

## Methods

### Samples

One hundred and four commercially available cinnamon samples were purchased in Austria, Belgium, Bulgaria, France, Germany, Greece, Italy, Malta, Serbia, Slovakia, Spain, Sri Lanka and the UK. Samples were bought at supermarkets of different chains and small retailers, to avoid the risk of all the samples belonging to the same batch/original producer. Six samples were sold as bulk and the remaining were pre-packaged and branded as registered trademarks. Fourteen samples were purchased on-line, all of them commercialised by registered trademarks.

According to the information provided on the labels, 44 samples were *C. verum* referred to as Ceylon cinnamon in this work, 4*C. burmanii*, 1*C. burmanii/cassia/loureiroi*, 1*C. aromaticum* and 11 cassia cinnamon, all of them referred to as Cassia cinnamon in this work; for the remaining 43 no information about botanical species was provided. Regarding the origin of the cinnamon, 22 were from Sri Lanka, 6 from Madagascar, 7 from Vietnam, 2 from India, 3 from Indonesia, 2 were a mixture of cinnamon from Sri Lanka and Indonesia and 1 indicated Tropics. The remaining samples either indicated “outside EU”, or 4 “non-EU”, or no information about the origin was provided.

Six samples from Madagascar were included in the study. Ceylon cinnamon from Madagascar seems to have a special chemical composition when compared with that of other origins, as reflected by the higher total ash contents accepted by the ISO standard (7.5% for Ceylon cinnamon from Madagascar, while for the remaining geographical origins the limit is 5%). For this reason, test materials labelled as coming from Madagascar were analysed but they were not further evaluated, and referred to in Table 2 and in Supplementary Table 1 as not evaluated (NE).

Seventy samples were ground (according to the labels 27 *C. verum*, 3 *C. burmanii*, 1 *C. burmanii/cassia/loureiroi*, 1 *C. aromaticum*, and 9 were labelled as cassia; no information on botanical species was provided for the remaining 29). The remaining 34 samples were commercialised as bark (17*C. verum* and 2 cassia, no information on botanical species was provided for the remaining 15).

Thirty-three samples were labelled as “organic”, referred to as BIO in this work, it was assumed that the remaining samples were issued of “conventional” agriculture, referred to in this work as NON-BIO.

All available information about the cinnamon samples included in this study is summarised in Supplementary Table 1.

All test samples were kept at room temperature and in sealed bags to prevent absorption of humidity. For the cinnamon samples purchased in sticks, milling was applied to produce powder for testing.

### Materials and methods for determination of total ash by TGA

The ash content of the cinnamon test materials was determined with a ThermoGravimetric Analyzer (Mettler Toledo). Test materials were measured in duplicate with test portion sizes ranging from 35 to 64 mg. The test portions were heated in an oxidative atmosphere to 550 °C and held at that temperature until the weight of the residue stayed constant. The weight % of the residue was reported as the ash content.

### Materials and methods for analyses by EDXRF

The elemental composition (Na, Mg, Al, Si, P, Cl, S, K, Ca, Ti, Cr, Mn, Fe, Ni, Cu, Zn, As, Br, Rb, Sr, Zr, Cd, Ba, Pb, V, Co, Se, Mo, Sn and, Sb) of the cinnamon samples was determined by EDXRF, using an Epsilon 4 spectrometer (PANalytical, Almelo, The Netherlands). The instrument is equipped with a Silver (Ag) anode X-ray tube with a Beryllium (Be) side window (50 µm thickness) and a silicon drift detector SDD30. The maximum high tension, anode current and power are 50 kV, 3 mA and 15 W, respectively. The system has six filters (Cu-500, Ti, Al-50, Al-200, Cu-300 and Ag, with thickness of 500, 7, 50, 200, 300 and 100 µm, respectively) that generate the excitation X-rays. The selection of the appropriate filter depends on the element to be analysed and was done according to the recommendations of the instrument manufacturer. The system is air-cooled and does not require the use of liquid nitrogen. The instrument was calibrated every month with the CRM INCT-OBTL-5, Oriental Basma Tobacco Leaves (Department of Analytical Chemistry, Institute of Nuclear Chemistry and Technology) correcting in this way the normal drift of the instrument. The performance of the EDXRF spectrometer was checked monthly with the CRM INCT-PVTL-6, Polish Virginia Tobacco Leaves (Department of Analytical Chemistry, Institute of Nuclear Chemistry and Technology). The ED-XRF was controlled by the PANalytical Epsilon 4 Software, Version 2.0 N/ICSW2.11.

The EDXRF method was validated using an approach previously described^35^. Summarising, a single calibration curve covering different types of organic matrices was built for each element analysed using the Certified Reference Materials (CRM) and Reference Materials (RM) listed in Supplementary Table 2. Those CRMs and RMs for which the ratio between the calculated and the certified or indicative mass fraction was outside the range 70–130%, were left out of the calibration curve.

To evaluate the accuracy, trueness, and precision, of the method, CRMs and RMs indicated in Supplementary Table 2 were used. One pellet of each of the CRM/RM was measured in triplicate in three different measurement sessions spread over three days. The results obtained were used to evaluate the within and between-day variation. The intermediate precision calculated with Analysis of Variance (ANOVA), confidence interval 95%, using the software Statistica (TIBCO, version 13.5.0.17), was taken as expanded uncertainty (*k* = 2).

The fit-for-purpose of the trueness was evaluated with z-scores^36^, as described in detail elsewhere^35^.

The limits of quantification (LOQ) for the different elements, were set to the lowest mass fraction in the calibration curve for which satisfactory results were obtained with an intermediate precision ≤25%.

No full validation could be conducted for the elements Al, Ba, Br, Si, Ti, and Zr because the few CRMs/RMs used in the study with certified/reference mass fractions for those elements had to be used to construct the calibration curves and none was left for the trueness study. The LOQ, intermediate precision and the working range provided in Table 1 for those elements are just indicative and based on the calibration curves constructed with a limited number of CRMs/RMs. Only two or three CRMs/RMs had certified/reference mass fractions for V, Co, Sn, and Sb; for that reason, the LOQ, intermediate precision and working range could not be determined. The mass fractions obtained were used as semi-quantitative information for modelling purposes.

### Materials and methods for determination of volatile compounds by HS-GC-MS

The selection of compounds to be included in the quantitative study was based on previous semi-quantitative research^18^.

The following standards α-pinene (≥97%), D-limonene (≥95%), benzyl alcohol (≥99%), camphor (2-bornanone) (≥98%), hydrocinnamaldehyde (3-phenylpropanal) (≥95%), anethole (4-Allylanisole) (≥98%), cinnamaldehyde (≥99%), eugenol (≥99%), β-caryophyllene (purity not specified), coumarin (99 + %), cinnamyl acetate (≥97%), eugenol acetate (≥98%), caryophyllene oxide (≥95%), benzyl benzoate (≥99%) and 2-methoxy-4-propylphenol (≥99%) were used for calibration.

Stock standard solutions were prepared by dissolving weighted amounts of the standards in acetonitrile at 10, 50, 100, 500 and 1000 mgL^−1^, except for cinnamaldehyde, for which the concentrations of stock standard solutions were 100, 500, 1000, 10,000, 50,000 and 100,000 mgL^−1^.

The calibration samples were prepared by weighing aliquots of 10 μL of stock standard solutions and 10 μL of internal standard solutions into 20 mL screw top headspace vials. For cinnamaldehyde calibration, 20 μL of internal standard solutions were added instead.

Cinnamon sample preparation was performed by weighing 50 mg in 20 mL screw top headspace vials and spiking the sample with 10 μL internal standard solution (2-methoxy-4-propylphenol). The analysis of cinnamaldehyde required a dedicated method. Therefore, a sample replicate was prepared by weighing 50 mg cinnamon and spiking with 20 μL of internal standard solution.

The samples were incubated at 150 °C for 15 min in a ThermoPal autosampler (PAL COMBI-xt). An aliquot of 1 mL headspace gas was injected into a Trace1310 GC by means of a gas syringe kept at 150 °C. The injector was kept at 200 °C and the injection was performed with a split ratio of 10 (Topaz Split liner 1 × 6.3 × 78 mm). The chromatographic separation was performed on a DB-5MS capillary column (30-m length, 0.25-mm inner diameter, 0.25-μm film thickness) with helium carrier gas at 1 ml L^−1^. The GC oven was kept at 50 °C for 2 min and then ramped at 5 °C/min to 300 °C. Mass spectra were acquired with a TSQ Quantum XLS Ultra mass spectrometer scanning the mass range 45–450 with scan time of 0.2 s.

Cinnamaldehyde analysis was performed with a dedicated method. The vials were incubated at 150 °C for 15 min and 250 μL of headspace were injected into the GC, with a split ratio of 100. The oven temperature was kept at 50 °C for 2 min and then ramped to 300 °C at 30 °C/min. Mass spectra were acquired at the same conditions as the other volatile compounds.

A cinnamon mix containing 1 g of 52 cinnamon samples was prepared. A repeatability study was performed by analysing the cinnamon mix in three different days. In each one of the three days the analysis was performed in triplicate. The intermediate precision of the method calculated from the within- and between-pellet ANOVA, was taken as the expanded uncertainty (confidence interval 95%, *k* = 2) associated to the results.

LODs and LOQs (Table 1), were estimated based on the analysis of ten blanks. For each compound, the average peak area of the ten blanks was compared to that of the lowest calibration point to calculate the LOD. LOQs were estimated as three times the LOD levels.

### Materials and methods for qPCR analyses

DNA extraction, optimised for cinnamon bark^37^, was performed manually using a CTAB extraction buffer and included cleaning steps with chloroform/isoamyl alcohol (24/1 v/v) combined with 3 M sodium acetate (pH 5.2). DNA was precipitated overnight at −20 °C with isopropanol and washed thrice with ethanol 70% v/v before resuspension in the elution buffer. The DNA extract was quantified using a Qubit 4 fluorometer (Invitrogen) with Broad Range chemistry. Purity was assessed with a NanoDrop Eight spectrophotometer (ThermoFisher Scientific).

Quantitative real-time PCR (qPCR) was used to screen the presence of foreign species in the commercial samples purchased in powder. qPCR amplification was carried out in a 20 μL reaction volume using 10 μL PowerUp™ SYBR™ Green Master Mix (Applied Biosystems), 400 nM of each forward and reverse primer and 25 ng of extracted DNA (whenever possible) as template. For each primer pair, a non-template control consisting of 25 ng of pure Ceylon cinnamon DNA was included. The amplifications were performed with a QuantStudio 7 qPCR system (Applied Biosystems) in 96-well plates. Primer pairs were tested for cross-reactivity with other species by qPCR using SYBR™ Green chemistry. The investigated targets are given in Supplementary Table 3, some were selected based on information existing in the literature^38–41^.

### Multivariate analysis

Multivariate analysis of the volatile and elemental mass fractions of the cinnamon samples was done with the software SIMCA® version 17 (Umetrics, Malmö, Sweden)^42^. Principal component analysis (PCA), a non-supervised multivariate analysis tool that reduces the amount of variables (volatile and elemental mass fractions) into a small number of non-correlated principal components, was used to visualise clusters in function of their botanical variety, Ceylon and Cassia cinnamon, and to detect outliers. The only pre-processing applied was normalisation of the concentrations by unit-variance scaling. The amount of principal components was always set to three to avoid overfitting.

## Supplementary information

Supplementary Information

## Data Availability

The datasets generated and/or analysed during the current study are not publicly available due to confidentiality issues but are available from the corresponding author on reasonable request.
