# Supplementary material for: High rate of safety and fraud issues in commercially available cinnamon
Source: NPJ Sci Food. 2025 Jul 5;9:125. doi: 10.1038/s41538-025-00485-w (PMC12228808; doi:10.1038/s41538-025-00485-w)
Supplement: Supplementary file 1 — Supplementary Information [file 41538_2025_485_MOESM1_ESM.pdf]

Supplementary Table 1: Summary of information about the test samples analysed, the decisions based on the results of the different analytical techniques and the final decision. Empty cells in the decisions for the different techniques indicates that the sample was not suspicious. The following codes are used to indicate the results of each analytical techniques: S: Suspicious, NS: Non suspicious, NE: Not evaluated, S*: Lack of quality (high ash content), S?: Statistical outliers due to levels of contamination that could be due to cross-contamination.

Sample id	Label variety	BIO/NON BIO	Purchase form	Origin	S/NS (TGA)	S/NS (ED-XRF)	S/NS (HS-GC-MS)	S/NS (qPCR)	Final conclusion
Cin001	?	NON BIO	Ground	??					NS
Cin002	?	NON BIO	Ground	??					NS
Cin003	?	NON BIO	Ground	??					NS
Cin004	?	NON BIO	Ground	??					NS
Cin005	?	BIO	Ground	??	S	S	S		S
Cin006	?	NON BIO	Ground	??					NS
Cin007	?	NON BIO	Ground	??					NS
Cin008	?	NON BIO	Ground	??					NS
Cin009	?	NON BIO	Ground	??					NS
Cin010	?	NON BIO	Ground	??		S	S		S
Cin011	Ceylon	BIO	Ground	Madagascar					NE
Cin012	Ceylon	BIO	Ground	Sri Lanka		S			S?
Cin013	?	BIO	Ground	NON EU					NS
Cin014	Ceylon	BIO	Ground	Madagascar					NE
Cin015	Burmanni	BIO	Ground	??					NS
Cin016	Aromaticum	BIO	Ground	??	S				S*
Cin017	?	NON BIO	Ground	Ceylon and Indonesia		S	S		S
Cin018	Ceylon	BIO	Ground	NON EU		S	S	S	S
Cin019	Ceylon	BIO	Ground	NON EU		S	S		S
Cin020	Ceylon	BIO	Ground	NON EU		S	S		S

Sample id	Label variety	BIO/NON BIO	Purchase form	Origin	S/NS (TGA)	S/NS (ED-XRF)	S/NS (HS-GC-MS)	S/NS (qPCR)	Final conclusion
Cin021	Ceylon	NON BIO	Ground	??	S	S	S		S
Cin022	Ceylon	NON BIO	Ground	??	S	S	S		S
Cin023	Cassia	NON BIO	Ground	??					NS
Cin024	Cassia	BIO	Ground	Vietnam					NS
Cin025	Ceylon	NON BIO	Ground	??	S	S	S		S
Cin026	?	NON BIO	Ground	Vietnam		S	S		S
Cin027	Burmanni	NON BIO	Ground						NS
Cin028	Burmanni	BIO	Ground						NS
Cin029	Ceylon	NON BIO	Ground	??	S	S	S		S
Cin029 batch 2	Ceylon	NON BIO	Ground	??	S	S	S		S
Cin030	Cassia	NON BIO	Ground						NS
Cin030 batch 2	Cassia	NON BIO	Ground			S	S	S	S
Cin031	?	NON BIO	Ground	Sri Lanka					NS
Cin032	Ceylon	NON BIO	Stick	Sri Lanka				not analysed	NS
Cin033	Ceylon	NON BIO	Stick	Sri Lanka	S	S		not analysed	S*
Cin034	Ceylon	NON BIO	Stick	Sri Lanka		S		not analysed	S?
Cin035	Ceylon	BIO	Stick	Sri Lanka	S			not analysed	S*
Cin036	Ceylon	BIO	Stick	Sri Lanka	S			not analysed	S*
Cin037	Ceylon	BIO	Stick	??	S			not analysed	S*
Cin038			Stick		S	S	S	not analysed	S
Cin039	Ceylon	NON BIO	Stick	Sri Lanka	S	S	S	not analysed	S
Cin040	Ceylon	BIO	Ground	??		S	S		S
Cin041	Ceylon	NON BIO	Ground	Sri Lanka				S	S
Cin042	Ceylon	NON BIO	Stick	Sri Lanka	S	S		not analysed	S*
Cin043	Ceylon	BIO	Ground	??	S	S	S		S
Cin044	Ceylon	NON BIO	Ground	Madagascar					NE
Cin045	Ceylon	NON BIO	Ground	Sri Lanka		S	S		S
Cin046	Ceylon	BIO	Ground	India		S	S		S

Sample id	Label variety	BIO/NON BIO	Purchase form	Origin	S/NS (TGA)	S/NS (ED-XRF)	S/NS (HS-GC-MS)	S/NS (qPCR)	Final conclusion
Cin047	Ceylon	BIO	Stick	??		S	S	not analysed	S
Cin048	Ceylon	BIO	Stick	Sri Lanka				not analysed	NS
Cin049	Ceylon	BIO	Ground	??		S	S		S
Cin050	Ceylon	NON BIO	Ground	Sri Lanka		S	S		S
Cin051	Ceylon	NON BIO	Ground	Sri Lanka		S	S		S
Cin052	Cassia	NON BIO	Stick	Indonesia				not analysed	NS
Cin053 batch1	?	NON BIO	Ground	Ceylan and Indonesia		S	S		S?
Cin053 batch2	?	NON BIO	Ground	Sri Lanka (Ceylan) and Indonesia		S	S	S	S?
Cin054 batch1	Ceylon	NON BIO	Stick	?	S	S		not analysed	S?
Cin054 batch2	Ceylon	NON BIO	Stick	?		S		not analysed	S?
Cin054 batch3	Ceylon	NON BIO	Stick	?	S		S?	not analysed	S?
Cin055	?	?	Ground	?				S	S
Cin056	?	NON BIO	Stick	Indonesia				not analysed	NS
Cin057	Ceylon	BIO	Ground	Sri Lanka		S	S		S
Cin058	Ceylon	BIO	Ground	Sri Lanka		S	S		S
Cin059	Ceylon	BIO	Stick	Sri Lanka				not analysed	NS
Cin060	?	NON BIO	Ground	?					NS
Cin061	?	NON BIO	Stick	?				not analysed	NS
Cin062	?	NON BIO	Stick	?				not analysed	NS
Cin063	?	NON BIO	Ground	Tropics		S			S
Cin064	burmanni/cassia/loureiroi	NON BIO	Ground	?					NS
Cin065	Ceylon	BIO	Ground	Sri Lanka	S	S	S	S	S*
Cin066	?	NON BIO	Stick	Vietnam	S		S	not analysed	S*
Cin067	?	NON BIO	Ground	Vietnam		S			S
Cin068	?	NON BIO	Ground	Outside EU		S			S
Cin069	Ceylon	BIO	Stick	Outside EU		S?		not analysed	NS
Cin070	Cassia	BIO	Ground	Vietnam	S	S			S

Sample id	Label variety	BIO/NON BIO	Purchase form	Origin	S/NS (TGA)	S/NS (ED-XRF)	S/NS (HS-GC-MS)	S/NS (qPCR)	Final conclusion
Cin071	Ceylon	BIO	Ground	Outside EU	S	S	S		S
Cin072	Cassia	?	Ground	?		S			S
Cin073	Ceylon	?	Ground	?		S	S		S
Cin074	Cassia	BIO	Ground	India		S	S		S
Cin075	Ceylon	BIO	Ground	Madagascar					NE
Cin076	Ceylon	BIO	Ground	Sri Lanka		S?	S		S?
Cin077	Ceylon	BIO	Stick	Sri Lanka				not analysed	NS
Cin078	?	NON BIO	Stick	Indonesia				not analysed	NS
Cin079	?	NON BIO	Stick	?				not analysed	NS
Cin080	?	NON BIO	Ground	?					NS
Cin081	?	NON BIO	Stick	?	S		S	not analysed	S
Cin082	?	NON BIO	Ground	?			S		NS
Cin083	Cassia	NON BIO	Ground	?		S			NS
Cin084	?	NON BIO	Stick	Vietnam		S		not analysed	NS
Cin085	?	NON BIO	Ground	Vietnam	S	S			S
Cin086	?	NON BIO	Ground	?		S			S
Cin093	?	NON BIO	Stick	?	S		S?	not analysed	NS
Cin094	?	NON BIO	Ground	?			S	S	S
Cin095	?	NON BIO	Stick	?			S?	not analysed	NS
Cin096	?	NON BIO	Ground	?					NS
Cin097	?	NON BIO	Stick	China				not analysed	NS
Cin098	Cassia	NON BIO	Ground	?	S	S			S*
Cin099	Cassia	NON BIO	Stick	?				not analysed	NS
Cin100	Ceylon	NON BIO	Stick	?		S	S?	not analysed	S
Cin101	?	NON BIO	Stick	?		S		not analysed	NS
Cin102	?		Ground		S				NS
Cin 103	?		Stick	Sri Lanka	S	S?		not analysed	S
Cin 104	?		Stick	Sri Lanka	S	S	S?	not analysed	S

Sample id	Label variety	BIO/NON BIO	Purchase form	Origin	S/NS (TGA)	S/NS (ED-XRF)	S/NS (HS-GC-MS)	S/NS (qPCR)	Final conclusion
Cin 105			Stick	Madagascar				not analysed	NE
Cin 106			Ground	Madagascar		S	S		S

Supplementary Table 2: CRMs and RMs used for calibration purposes and in accuracy studies.

Calibration curve		Accuracy studies	
**CRM/RM**	**Matrix**	**CRM/RM**	**Matrix**
BCR 129	Hay powder	BOVM-1	Bovine muscle
BCR 191	Brown bread	DOLT-5	Dogfish liver
BCR 402	White clover	KINO-1	Kinoa flour
BCR 414	Plankton	REDS-1	Hard red spring wheat flour
BCR 482	Lichen	SPIN-1	Spinach
BCR 679	White cabbage	TORT-3	Lobster hepatopancreas
IRMM 804	Rice flour	VITA-1	Multivitamin
ERBM-CD 281	Rye grass		
ERM-BB 422	Fish muscle		
ERM-BB 184	Bovine muscle		
ERM-BB 185	Bovine liver		
ERM-BB 186	Pig kidney		
IMEP 119	Vegetable feed		
IRMM PT-43	Rice flour		
BRAN-1	Corn bran		
DUWF-1	Durum wheat flour		
DORM-5	Fish protein		
NIST 1548a	Mixed diet		
NIST 1549a	Whole milk powder		
NIST 1566b	Oyster tissue		
NIST 1568b	Rice flour		
NIST 1570a	Spinach leaves		
NIST 1573a	Tomato leaves		
NIST 1575a	Pine needles		
NIST 3234	Soy flour		
NIST 3252	Protein drink mix		
NIST 3287	Blueberry		
NIST 3290	Dry cat food		
OBTL-5	Tobacco		
PVTL-6	Tobacco		
IAEA 336	Lichen		
IAEA 359	Cabbage		
IAEA 392	Algae		
IAEA 413	Algae		
NMIJ 7405-a	Seaweed (Hijiki)		
RT3	Tobacco		
RT5	Tobacco		
AJJA-17	Tobacco		

Supplementary Table 3: qPCR primers used as targets in the q-PCR method used to screen the presence of foreign species in cinnamon.

Species	Target	Primers (fw / rv), 5'3'	Size (bp)	Reference
Allium cepa	Maturase K	CACGAATACCATAATTGGAATAATCTTTA<br>AGCACTAATAAAATTTCGCACTCAAA	134	[36]
Allium sativum	Alliinase	GCCTCATTACAGCCCAATCA<br>CATCCTTTATCAACGCCCAC	112	[37]
Arachis hoehnei / hypogaea	Arah3 C	TGTTTGTCCCTCACTACAACA<br>GAAGCTCCTCGTCGTACA	114	[34]
Brassica spp.	Cruciferin	CAGCTCAACAGTTTCCAAACGA<br>CGACCAGCCTCAGCCTTAAG	85	[37]
Calendula officinalis	OPB10	AAGGTTTAGGCGGGATCCTC<br>AGCGTGTTTCCGGCTTTTAC	116	[37]
Capsicum spp.	Pun1 (acyltransferase)	ACCTCGTCTACCACGAAATGCC<br>TGCCACCTCAACTTCCTTCCT	124	[37]
Coffea spp.	ATP-dependent Clp protease	TTCCGAAGTCCTGGAGAG<br>CGGAGGATATCTCAATCG	114	[35]
Melilotus spp.	GA3ox1	GTGCCGACGATACAAATGAC<br>ACCCTTTCATTCTTCCAAGG	126	This study
Oryza sativa	Phospholipase D	TGGTGAGCGTTTTGCAGTCT<br>CTGATCCACTAGCAGGAGGTCC	68	[37]
Piper nigrum	Hydroxycinnamoyl transferase	GCCGCAGATTCTCAAGGA<br>CGAAGTCGCCGAAGTCAT	170	[37]
Sinapis alba	MADS D	TGAAAACTCTCTTCCCCTCTTAGG<br>ACAAATGCACACAAGACAGAGATATAGA	74	[37]
Syzygium aromaticum	Elongation factor 1a	TAGGGAGCATGCTTTGCTTG<br>GCCAGGAATTTACCTTGTTGC	77	This study
Trigonella foenum-graecum	Centromere CenH3	CCAGATACGACACTGACACGTA<br>CAAACCTATGTCGGTGTCTGA	144	This study
